# Retracted: Potentiated Osteoinductivity via Cotransfection with BMP-2 and VEGF Genes in Microencapsulated C2C12 Cells

**DOI:** 10.1155/2022/9781609

**Published:** 2022-05-25

**Authors:** BioMed Research International

*BioMed Research International* has retracted the article titled “Potentiated Osteoinductivity via Cotransfection with BMP-2 and VEGF Genes in Microencapsulated C2C12 Cells” [1] due to errors identified with the figures.

Following the publication of the article, concerns have been identified by the authors with figures of the article and also raised on Pubpeer [2]:

The concerns are related to Figures 1 and 2:

- The authors raised that in Figure 1(a), the image of ADSCs was incorrectly selected by them.
- In addition, Figure 2(b) 1w ADSCs, Figure 2(b) 4w SMSCs and Figure 2(c) 4w C2C12 were selected inappropriately.
- While assessing these concerns, it was also found that Figure 2(b) ADSCs at 1 week is the same as Figure 2(c) NIH/3 T3 at 1 week, with the contrast and exposure adjusted. This was not raised by the authors during their correction request.

The authors did not provide a satisfactory response. Hence, this article is being retracted with the agreement of the editorial board.

## References

[1] Shen Y., Qiao H., Fan Q., Zhang S., Tang T. (2015). Potentiated Osteoinductivity via Cotransfection with BMP-2 and VEGF Genes in Microencapsulated C2C12 Cells. *BioMed Research International*.

[2] Potentiated Osteoinductivity via Cotransfection with BMP-2 and VEGF Genes in Microencapsulated C2C12 Cells. https://pubpeer.com/publications/85E08C2D506807F9A1B40DE5FC3FA3.

